# Protein tyrosine nitration in higher plants grown under natural and stress conditions

**DOI:** 10.3389/fpls.2013.00029

**Published:** 2013-02-25

**Authors:** Francisco J. Corpas, José M. Palma, Luis A. del Río, Juan B. Barroso

**Affiliations:** ^1^Departamento de Bioquímica, Biología Celular y Molecular de Plantas, Estación Experimental del Zaidín, Consejo Superior de Investigaciones CientíficasGranada, Spain; ^2^Grupo de Señalización Molecular y Sistemas Antioxidantes en Plantas, Unidad Asociada al Consejo Superior de Investigaciones Científicas (EEZ), Área de Bioquímica y Biología Molecular, Universidad de JaénJaén, Spain

**Keywords:** nitric oxide, nitroproteome, peroxynitrite, reactive nitrogen species (RNS), tyrosine nitration

## Abstract

Protein tyrosine nitration is a post-translational modification (PTM) mediated by reactive nitrogen species (RNS) that is linked to nitro-oxidative damages in plant cells. During the last decade, the identification of proteins undergoing this PTM under adverse environmental conditions has increased. However, there is also a basal endogenous nitration which seems to have a regulatory function. The technological advances in proteome analysis have allowed identifying these modified proteins and have shown that the number and identity of the nitrated proteins change among plant species, analysed organs and growing/culture conditions. In this work, the current knowledge of protein tyrosine nitration in higher plants under different situations is reviewed.

## Introduction

Protein tyrosine nitration is a post-translational modification (PTM) mediated by nitric oxide-derived molecules. It is the result of the addition of a nitro (−NO_2_) group to one of two equivalent ortho carbons in the aromatic ring of tyrosine residues (Gow et al., 2004). This process can alter protein function because the incorporation of this nitro group into the aromatic ring lowers the pKa of the phenolic group from 10.1 of the tyrosine to 7.2 in the nitrotyrosine (Sokolovsky et al., 1967; Abello et al., 2009). This provokes both steric and electronic perturbations that affect Tyr's capacity to function in electron-transfer reactions and to maintain protein conformation (van der Vliet et al., 1999). Tyrosine nitration can change the function of the protein in several ways: function gain; no effect on function; and inhibition of function, the latter being the most common consequence of tyrosine nitration (Greenacre and Ischiropoulos, 2001; Radi, 2004). On the other hand, tyrosine nitration may influence many signal transduction pathways because this modification prevents phosphorylation of tyrosine and consequently affects one regulatory mechanism (Galetskiy et al., 2011).

**Figure 1:**
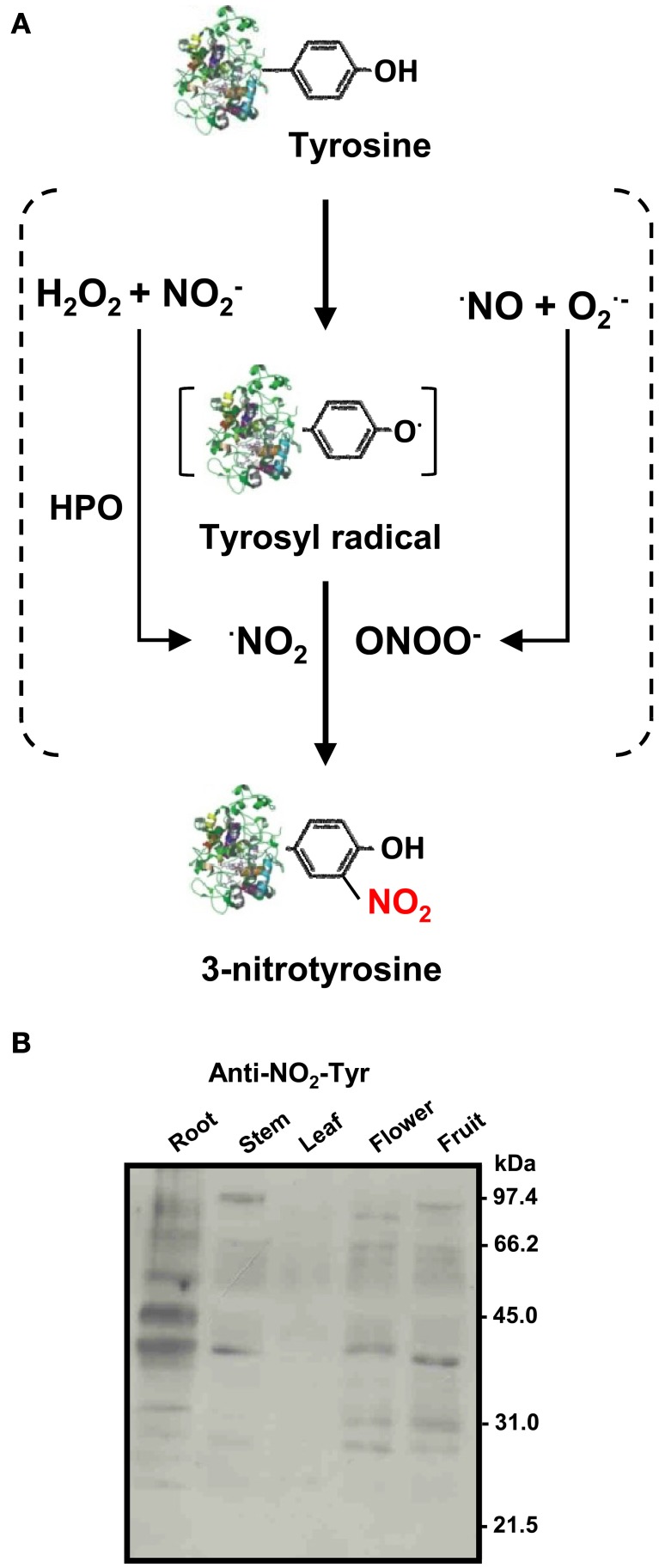
**(A)** Biochemistry of protein tyrosine nitration (NO_2_-Tyr). Tyrosine nitration involves two steps: oxidation of the phenolic ring of tyrosine to tyrosyl radical (Tyr^·^) and addition of ^·^NO_2_ to the Tyr^·^ by a nitrating agent. The Tyr radical can be produced by several one-electron oxidants such as ^·^NO_2_, ^·^OH, or CO^·−^_3_. There are two main nitrating reactions: (1) by peroxynitrite (ONOO^−^) which is formed by the quick reaction between nitric oxide (^·^NO) and superoxide (O^·−^_2_) radicals; and (2) By ^·^NO_2_ produced by reaction of hydrogen peroxide (H_2_O_2_) and nitrite (NO^−^_2_) in the presence of hemoperoxidase (HPO) (Souza et al., 2008). **(B)** Representative immunoblot showing the pattern of protein tyrosine nitration (NO_2_-Tyr) in different organs (root, stem, leaf, flower, and fruit) of pea plants after 71 days of growth under optimal conditions. The numbers on the right side of the immunoblots indicate the relative molecular masses of the protein markers (reproduced from Corpas et al., 2009).

The presence of nitrotyrosine has been considered as a footprint of the occurrence of the strong nitrating agent peroxynitrite (ONOO^−^), a reactive nitrogen species (RNS), which is formed usually under stress conditions by the chemical reaction between two radicals, nitric oxide (^·^NO) and superoxide anion (O^·−^_2_) (Ischiropoulos, 2003; Chaki et al., 2009a; Arasimowicz-Jelonek and Floryszak-Wieczorek, 2011). However, there is another potential mechanism involving a hemoperoxidase that, in the presence of hydrogen peroxide (H_2_O_2_) and nitrite (NO^−^_2_), can generate the radical nitrogen dioxide (^·^NO_2_) which acts as a nitrating agent (Souza et al., 2008) (Figure 1A). A relevant aspect is that tyrosine nitration is not a random process because not all Tyr residues are susceptible to nitration which depends on their accessibility to the solvent. Tyrosine has a hydropathy index of −1.3 being a mildly hydrophilic amino acid and, therefore, most likely exposed to the aqueous environment.

In most proteins, the number of tyrosine residues is around 3–4% out of the primary structure, but only few of these tyrosines may become preferentially nitrated. A good example is the human serum albumin which has 18 Tyr residues, and under *in vitro* nitration by peroxynitrite only 2 Tyr are susceptible of nitration. In animal organisms, the number of nitrotyrosine-containing proteins identified by proteomics approaches ranges from few proteins to over 100, depending on the organ (brain, heart, liver, etc.), subcellular compartment, and physiological conditions (normal or stress situations) (Abello et al., 2009).

## *In vivo* detection of protein targets of tyrosine nitration in plants

In higher plants, tyrosine nitration was initially detected using 1D gel electrophoresis followed by immunoblotting probed with antibodies against 3-nitrotyrosine. Thus, in tobacco leaf extracts and BY2 cell cultures it was observed that the exogenous application of nitrating agents such as peroxynitrite provoked a rise of immunoreactive proteins (Morot-Gaudry-Talarmain et al., 2002; Saito et al., 2006). Later, a similar behavior in the profile of nitrated proteins was observed in leaves of olive plants grown with 200 mM NaCl which induced both oxidative and nitrosative stresses (Valderrama et al., 2007). After detecting the proteins which underwent tyrosine nitration, the next step was to identity those proteins which were targets of this PTM. Thus, the combination of proteomic techniques (two-dimensional polyacryamide gel electrophoresis) followed by immunoblotting or immunoprecipitation, gel tryptic digestion and mass spectrometry have become powerful tools to develop nitroproteome studies in higher plants. Although there might be some technical problems like those due to the unspecific recognition of the antibody used against nitrotyrosine, the low number of nitrotyrosines in a given protein, the low abundance of the nitrotyrosine-containing proteins, and that the isolated protein occasionally does not match in the protein database, it must be pointed out that these approaches have allowed establishing a basic background of knowledge in this research area (Dekker et al., 2012). In any case, after a specific protein has been identified as a putative nitration target, a necessary additional step is to identify the nitration site(s) within the protein quaternary structure by MALDI-TOF MS and LC-MS/MS (Ytterberg and Jensen, 2010). Finally, *in vitro* analyses of the physiological effects of nitration on the specific proteins must be developed. Table 1 summarizes some of the identified nitrated proteins in higher plants where the locus for the nitrated tyrosine residue have been identified and the physiological effect of this PTM established.

**Table 1:** **Examples of proteins identified in higher plants which are targets of tyrosine nitration, and the effect of this PTM on their function**.

**Protein**	**Plant species**	**Subcellular localization**	**Effects**	**Identified nitrated Tyr**	**References**
*S*-adenosyl homocysteine hydrolase (SAHH)	Sunflower	Nucleus	Decreased activity	Tyr-448^a^	Chaki et al., 2009a
Ferredoxin–NADP reductase	Sunflower	Chloroplast	Decreased activity	ND	Chaki et al., 2011
Carbonic anhydrase (β-CA)	Sunflower	Chloroplast	Decreased activity	Tyr-205^a^	Chaki et al., 2013
PSBA(D1) of Photosystem II complex	*Arabidopsis*	Chloroplast	Disassembly of PSII dimers	Tyr-262^b^	Galetskiy et al., 2011
Methionine synthase	*Arabidopsis*	Cytosol	Decreased activity	Tyr-287^b^	Lozano-Juste et al., 2011
Glutamine synthetase	*Medicago truncatula*	Cytosol Chloroplast	Decreased activity	ND	Melo et al., 2011
O-acetylserine(thiol) lyase A1	*Arabidopsis*	Cytosol	Decreased activity	Tyr-302^b^	Álvarez et al., 2011
Glyceraldehyde-3-phosphate dehydrogenase	*Arabidopsis thaliana*	Cytosol Chloroplast	Decreased activity	ND	Lozano-Juste et al., 2011
NADP-isocitrate dehydrogenase	Pea	Cytosol	Decreased activity	Tyr-392^b^	Begara-Morales et al., 2013
α-Tubulin	Rice and tobacco cell cultures	Microtubules	Mitosis inhibition	ND	Jovanović et al., 2010

a In silico identification.

b Mass spectrometric techniques (LC-MS/MS).

ND: Not determined.

## Nitration and environmental stress situations

Tyrosine nitration has been mainly studied in plants under stress conditions and it is assumed that a rise in the protein tyrosine nitration is a reliable marker of nitro-oxidative stress (Corpas et al., 2007). A significant number of data in different organs and plant species support this idea since increases of some specific nitrated proteins under different abiotic and biotic stresses have been reported. Thus, this PTM event occurs in leaves from pea plants subjected to different abiotic stresses including low and high temperature (HT), continuous light, and high light intensity (Corpas et al., 2008), in olives leaves, in *Arabidopsis* roots and *Citrus* leaves under salinity stress (Valderrama et al., 2007; Corpas et al., 2009; Tanou et al., 2012), in sunflower hypocotyls infected by the pathogen *Plasmopara halstedii* (Chaki et al., 2009a), in *Prunus* genotypes under high bicarbonate and high pH (Cellini et al., 2011), in pepper leaves under low temperature (Airaki et al., 2012), in *Arabidopsis* seedlings under arsenic stress (Leterrier et al., 2012), and in *Lotus japonicus* roots and leaves exposed to water stress (Signorelli et al., 2013). However, there are different environmental conditions (hypoxia, UV radiation, ozone, etc.) where protein tyrosine nitration has not been studied yet.

The nitroproteome analysis under certain stress conditions has allowed the identification of the induced proteins. Thus, in *Arabidopsis* leaves, up to eight different proteins undergoing Tyr-nitration and mainly involved in photosynthesis were identified (Cecconi et al., 2009). After infection with an avirulent bacterial pathogen (*Pseudomonas syringae* pv. Tomato), a general rise in the expression of those nitrated proteins was observed. Besides, three new nitrated proteins were detected, although they could not be identified (Cecconi et al., 2009). High light conditions induced specific tyrosine nitration in the protein PSBA(D1) of PhotosystemII (PSII) which provoked a dissociation of the PSII dimers and PSII-LHCII supercomplexes and a possible subsequent degradation of damaged protein subunits (Galetskiy et al., 2011). The exposure of sunflower seedlings to HT caused both oxidative and nitrosative stress, and nitroproteome analysis showed an increase in 12 tyrosine-nitrated proteins compared to control plants, plus the detection of a newly nitrated protein, a carbonic anhydrase (CA) (Chaki et al., 2011). Among the tyrosine-nitrated proteins under HT stress, two of them were investigated in more detail, ferredoxin-NADP reductase (FNR) and CA, since both enzymes are involved in photosynthetic carbon assimilation, a process very sensitive to HT. Under HT stress the activities of FNR and CA were inhibited by 31% and 43%, respectively. This inhibition was corroborated under *in vitro* conditions, where their respective activities were determined in the presence of peroxynitrite as nitrating agent (Chaki et al., 2011, 2013).

Nitration can also provoke a loss of function as observed for different plant enzyme activities analyzed *in vitro* including ascorbate peroxidase and catalase (Clark et al., 2000), *S*-adenosyl homocysteine hydrolase (Chaki et al., 2009b), and O-acetylserine(thiol)lyase A1 activities (Álvarez et al., 2011).

## Nitration and plant development

As indicated above, tyrosine nitration has been associated with situations of nitro-oxidative stress. However, some data illustrate the existence of a physiological protein nitration, which is not directly related to specific adverse conditions. Thus, in sunflower seedlings grown under optimal conditions, nitroproteome analysis of hypocotyl samples allowed the identification of 21 nitrotyrosine-immunopositive proteins involved in photosynthesis, and antioxidative, ATP, carbohydrate, and nitrogen metabolisms (Chaki et al., 2009b). More recently, nitroproteome analysis of *Citrus* roots revealed 26 potential candidate proteins to nitration (Tanou et al., 2012). Moreover, comparison of the nitroproteomes from green and red mature pepper (*Capsicum annuum* L.) fruits allowed identifying, by 2D gel and immunoblot, the profile of nitrated proteins which changed from 21 immunoreactive spots to 31 during fruit ripening. This could mean that protein tyrosine nitration could be used as an indicator of the ripening process in fruits. In rice seedlings and in tobacco BY-2 suspension cells grown under normal conditions, tyrosine nitration of α-tubulin may inhibit cell division and consequently cell growth as mitosis is inhibited (Jovanović et al., 2010).

Recently, nitration analysis during development and senescence of different organs from 8, 12, 14, and 16-day-old (young), and 71-day-old (senescent) pea plants has shown that each organ has its own protein nitration pattern (Figure 1B). In the case of roots, it was observed that the intensity of nitrated proteins increased with root age. Roots of senescent pea plants contained 16 nitrotyrosine-immunoreactive proteins. Among the identified proteins, cytosolic NADP-isocitrate dehydrogenase, an enzyme involved in carbon and nitrogen metabolism, redox regulation, and response to oxidative stress, was studied to determine the effect of nitration during root senescence, a developmental stage which produced a significant decrease of its activity (Begara-Morales et al., 2013).

## Conclusions

In higher plants, there is a growing interest in the analysis of protein tyrosine nitration as well as the identification of *in vivo* nitrated proteins. These studies are difficult to accomplish since tyrosine nitration is a low-abundance PTM. For example, in animal organisms under inflammatory conditions the estimated frequency of tyrosine nitration is 1 out of 10,000 tyrosines (Radi, 2004). The available data in higher plants are generally based on 2D gel electrophoresis and immunoblotting, what have provided some specific nitroproteomes including 21 proteins in sunflower cotyledons (Chaki et al., 2009a), 16 proteins in pea roots (Begara-Morales et al., 2013), and 127 proteins in *Arabidopsis* whole seedlings (Lozano-Juste et al., 2011). The identification of specific nitroproteomes from different plant organs under natural and stress conditions will be an exciting challenge for incoming research. Additionally, the study of the nitroproteome at subcellular level can provide fundamental data on cell proteomics of plants under different conditions.

### Conflict of interest statement

The authors declare that the research was conducted in the absence of any commercial or financial relationships that could be construed as a potential conflict of interest.

## References

[B1] Abello N. Kerstjens H. A. Postma D. S. Bischoff R. (2009). Protein tyrosine nitration: selectivity, physicochemical and biological consequences, denitration, and proteomics methods for the identification of tyrosine-nitrated proteins. J. Proteome Res. 8, 3222–3238 10.1021/pr900039c 19415921

[B2] Airaki M. Leterrier M. Mateos R. M. Valderrama R. Chaki M. Barroso J. B. (2012). Metabolism of reactive oxygen species and reactive nitrogen species in pepper (*Capsicum annuum* L.) plants under low temperature stress. Plant Cell Environ. 35, 281–295 10.1111/j.1365-3040.2011.02310.x 21414013

[B3] Álvarez C. Lozano-Juste J. Romero L. C. García I. Gotor C. León J. (2011). Inhibition of *Arabidopsis* O-acetylserine(thiol)lyase A1 by tyrosine nitration. J. Biol. Chem. 86, 578–586 10.1074/jbc.M110.147678 21047785 PMC3013017

[B4] Arasimowicz-Jelonek M. Floryszak-Wieczorek J. (2011). Understanding the fate of peroxynitrite in plant cells–from physiology to pathophysiology. Phytochemistry 72, 681–688 10.1016/j.phytochem.2011.02.025 21429536

[B5] Begara-Morales J. C. Chaki M. Sánchez-Calvo B. Mata-Pérez C. Leterrier M. Valderrama R. (2013). Protein tyrosine nitration in pea roots during development and senescence. J. Exp. Bot. 64, [Epub ahead of print]. 10.1093/jxb/ert006 23362300 PMC3580824

[B6] Cecconi D. Orzetti S. Vandelle E. Rinalducci S. Zolla L. Delledonne M. (2009). Protein nitration during defense response in *Arabidopsis thaliana*. Electrophoresis 30, 2460–2468 10.1002/elps.200800826 19598157

[B7] Cellini A. Corpas F. J. Barroso J. B. Masia A. (2011). Nitric oxide content is associated with tolerance to bicarbonate-induced chlorosis in micropropagated *Prunus* explants. J. Plant Physiol. 168, 1543–1549 10.1016/j.jplph.2011.02.008 21507506

[B8] Chaki M. Carreras A. López-Jaramillo J. Begara-Morales J. C. Sánchez-Calvo B. Valderrama R. (2013). Tyrosine nitration provokes inhibition of carbonic anhydrase (β-CA) activity under high temperature stress. Nitric Oxide 29C, 30–33 10.1016/j.niox.2012.12.003 23266784

[B9] Chaki M. Valderrama R. Fernández-Ocaña A. M. Carreras A. Gómez-Rodríguez M. V. López-Jaramillo J. (2011). High temperature triggers the metabolism of *S*-nitrosothiols in sunflower mediating a process of nitrosative stress which provokes the inhibition of ferredoxin-NADP reductase by tyrosine nitration. Plant Cell Environ. 34, 1803–1818 10.1111/j.1365-3040.2011.02376.x 21676000

[B10] Chaki M. Valderrama R. Fernández-Ocaña A. M. Carreras A. López-Jaramillo J. Luque F. (2009a). Protein targets of tyrosine nitration in sunflower (*Helianthus annuus* L.) hypocotyls. J. Exp. Bot. 60, 4221–4234 10.1093/jxb/erp263 19717529

[B11] Chaki M. Fernández-Ocaña A. M. Valderrama R. Carreras A. Esteban F. J. Luque F. (2009b). Involvement of reactive nitrogen and oxygen species (RNS and ROS) in sunflower-mildew interaction. Plant Cell Physiol. 50, 265–279 10.1093/pcp/pcn196 19112080

[B12] Clark D. Durner J. Navarre D. A. Klessig D. F. (2000). Nitric oxide inhibition of tobacco catalase and ascorbate peroxidase. Mol. Plant Microbe Interact. 13, 1380–1384 10.1094/MPMI.2000.13.12.1380 11106031

[B13] Corpas F. J. Chaki M. Fernández-Ocaña A. Valderrama R. Palma J. M. Begara-Morales J. C. (2008). Metabolism of reactive nitrogen species in pea plants under abiotic stress conditions. Plant Cell Physiol. 49, 1711–1722 10.1093/pcp/pcn144 18801763

[B14] Corpas F. J. Chaki M. Leterrier M. Barroso J. B. (2009). Protein tyrosine nitration: a new challenge in plants. Plant Signal. Behav. 4, 1–4 10.4161/psb.4.10.9466 19826215 PMC2801353

[B15] Corpas F. J. del Río L. A. Barroso J. B. (2007). Need of biomarkers of nitrosative stress in plants. Trends Plant Sci. 12, 436–438 10.1016/j.tplants.2007.08.013 17826297

[B16] Dekker F. Abello N. Wisastra R. Bischoff R. (2012). Enrichment and detection of tyrosine-nitrated proteins. Curr. Protoc. Protein Sci. 69, 14.13.1–14.13.19 10.1002/0471140864.ps1413s69 22851496

[B17] Galetskiy D. Lohscheider J. N. Kononikhin A. S. Popov I. A. Nikolaev E. N. Adamska I. (2011). Phosphorylation and nitration levels of photosynthetic proteins are conversely regulated by light stress. Plant Mol. Biol. 77, 461–473 10.1007/s11103-011-9824-7 21901528

[B18] Gow A. J. Farkouh C. R. Munson D. A. Posencheg M. A. Ischiropoulos H. (2004). Biological significance of nitric oxide-mediated protein modifications. Am. J. Physiol. Lung Cell Mol. Physiol. 287, L262–L268 10.1152/ajplung.00295.2003 15246980

[B19] Greenacre S. A. Ischiropoulos H. (2001). Tyrosine nitration: localisation, quantification, consequences for protein function and signal transduction. Free Radic. Res. 34, 541–581 11697033 10.1080/10715760100300471

[B20] Ischiropoulos H. (2003). Biological selectivity and functional aspects of protein tyrosine nitration. Biochem. Biophys. Res. Commun. 305, 776–783 10.1016/S0006-291X(03)00814-3 12763060

[B21] Jovanović A. M. Durst S. Nick P. (2010). Plant cell division is specifically affected by nitrotyrosine. J. Exp. Bot. 61, 901–909 10.1093/jxb/erp369 20018903 PMC2814120

[B22] Leterrier M. Airaki M. Palma J. M. Chaki M. Barroso J. B. Corpas F. J. (2012). Arsenic triggers the nitric oxide (NO) and *S*-nitrosoglutathione (GSNO) metabolism in *Arabidopsis*. Environ. Pollut. 166, 136–143 10.1016/j.envpol.2012.03.012 22504427

[B23] Lozano-Juste J. Colom-Moreno R. León J. (2011). *In vivo* protein tyrosine nitration in *Arabidopsis thaliana*. J. Exp. Bot. 62, 3501–3517 10.1093/jxb/err042 21378116 PMC3130175

[B24] Melo P. M. Silva L. S. Ribeiro I. Seabra A. R. Carvalho H. G. (2011). Glutamine synthetase is a molecular target of nitric oxide in root nodules of *Medicago truncatula* and is regulated by tyrosine nitration. Plant Physiol. 157, 1505–1517 10.1104/pp.111.186056 21914816 PMC3252174

[B25] Morot-Gaudry-Talarmain Y. Rockel P. Moureaux T. Quilleré I. Leydecker M. T. Kaiser W. M. (2002). Nitrite accumulation and nitric oxide emission in relation to cellular signaling in nitrite reductase antisense tobacco. Planta 215, 708–715 10.1007/s00425-002-0816-3 12244435

[B26] Radi R. (2004). Nitric oxide, oxidants, and protein tyrosine nitration. Proc. Natl. Acad. Sci. U.S.A. 101, 4003–4008 10.1073/pnas.0307446101 15020765 PMC384685

[B27] Saito S. Yamamoto-Katou A. Yoshioka H. Doke N. Kawakita K. (2006). Peroxynitrite generation and tyrosine nitration in defense responses in tobacco BY-2 cells. Plant Cell Physiol. 47, 689–697 10.1093/pcp/pcj038 16556649

[B28] Signorelli S. Corpas F. J. Omar Borsani O. Barroso J. B. Monza J. (2013). Water stress induces a differential and spatially distributed nitro-oxidative stress response in roots and leaves of *Lotus japonicus*. Plant Sci. 201–202, 137–146 10.1016/j.plantsci.2012.12.004 23352412

[B29] Sokolovsky M. Riordan J. F. Vallee B. L. (1967). Conversion of 3-nitrotyrosine to 3-aminotyrosine in peptides and proteins. Biochem. Biophys. Res. Commun. 27, 20–25 10.1016/S0006-291X(67)80033-0 6048236

[B30] Souza J. M. Peluffo G. Radi R. (2008). Protein tyrosine nitration -functional alteration or just a biomarker? Free Radic. Biol. Med. 45, 357–366 10.1016/j.freeradbiomed.2008.04.010 18460345

[B31] Tanou G. Filippou P. Belghazi M. Job D. Diamantidis G. Fotopoulos V. (2012). Oxidative and nitrosative-based signaling and associated post-translational modifications orchestrate the acclimation of citrus plants to salinity stress. Plant J. 72, 585–599 10.1111/j.1365-313X.2012.05100.x 22780834

[B32] Valderrama R. Corpas F. J. Carreras A. Fernández-Ocaña A. Chaki M. Luque F. (2007). Nitrosative stress in plants. FEBS Lett. 581, 453–461 10.1016/j.febslet.2007.01.006 17240373

[B33] van der Vliet A. Eiserich J. P. Shigenaga M. K. Cross C. E. (1999). Reactive nitrogen species and tyrosine nitration in the respiratory tract: epiphenomena or a pathobiologic mechanism of disease? Am. J. Respir. Crit. Care Med. 160, 1–9 10390372 10.1164/ajrccm.160.1.9807044

[B34] Ytterberg A. J. Jensen O. N. (2010). Modification-specific proteomics in plant biology. J. Proteomics 73, 2249–2266 10.1016/j.jprot.2010.06.002 20541636

